# Genome Selection and Genome-Wide Association Analyses for Litter Size Traits in Large White Pigs

**DOI:** 10.3390/ani15121724

**Published:** 2025-06-11

**Authors:** Yifeng Hong, Xiaoyan He, Dan Wu, Jian Ye, Yuxing Zhang, Zhenfang Wu, Cheng Tan

**Affiliations:** 1 College of Animal Science and National Engineering Research Center for Breeding Swine Industry, South China Agricultural University, Guangzhou 510642, China; hongyf0504@163.com; 2 State Key Laboratory of Swine and Poultry Breeding Industry, Guangzhou 510640, China; 3 National Engineering Research Center for Breeding Swine Industry, WENS Foodstuff Group Co., Ltd., Yunfu 527400, China; elainecn@163.com (X.H.); 15013019352@163.com (D.W.); jye1992@126.com (J.Y.); zhangyuxing1998@163.com (Y.Z.); 4 Yunfu Branch, Guangdong Laboratory for Lingnan Modern Agriculture, Yunfu 527300, China

**Keywords:** litter size traits, Large White, genetic parameters, GS, ssGWAS

## Abstract

(1) Background: Litter size traits are critical for pig breeding efficiency but pose challenges due to low heritability and sex-limited influences. This study aimed to elucidate the genetic architecture and identify candidate genes for these traits in Large White pigs using genomic selection (GS) and genome-wide association analyses (GWAS). (2) Methods: This study utilized phenotypic data from nine litter size traits in Large White sows. Genotyping-by-sequencing (GBS) was performed to obtain genotype data, retaining 153,782 high-quality SNPs after quality control. Genetic evaluation was conducted using single-step genomic best linear unbiased prediction (ssGBLUP), with genetic parameters (heritability and genetic correlations) estimated via an animal model (repeatability model). To assess prediction accuracy, 10-fold cross-validation was employed to compare traditional BLUP with ssGBLUP. Furthermore, a single-step genome-wide association study (ssGWAS) integrated genomic information and pedigree-based relationship matrices to screen for significant SNPs associated with litter size traits across the genome. Functional analysis of key candidate genes was subsequently conducted based on ssGWAS results. (3) Results: Heritabilities for litter traits ranged from 0.01 to 0.06. ssGBLUP improved genomic prediction accuracy by 6.38–13.33% over BLUP. Six genomic windows explaining 1.07–1.77% of genetic variance were identified via ssGWAS, highlighting *GPR12* on SSC11 as a key candidate gene linked to oocyte development. (4) Conclusions: This study demonstrates the efficacy of ssGBLUP for low-heritability traits and identifies GPR12 as a pivotal gene for litter size. Prioritizing NHB and LBWT in breeding programs could enhance genetic gains while mitigating adverse effects on piglet health. These findings advance genomic strategies for improving reproductive efficiency in swine.

## Simple Summary

Increasing the yield of healthy piglets per litter is economically important for pig production, yet constrained by biological factors affecting sow reproductive capacity. This study investigated genetic solutions using the Large White breed, known for its high reproductive performance. We recorded nine litter traits across 2096 sows, including total births and piglet weights. Advanced DNA analysis identified 153,782 genetic variations linked to these traits. A new breeding method improved sow productivity predictions by 6–13% compared to conventional approaches. Six genomic regions influencing litter size were discovered, notably the GPR12 as a pivotal gene for litter size. These findings advance genomic strategies for improving reproductive efficiency in swine.

## 1. Introduction

Litter size is a crucial breeding trait for pig production efficiency and profitability. Improving the total number of births and the number of healthy births in pigs has long been a primary goal for breeders. However, genetic improvement of litter size is challenging due to its low heritability and the significant impact of sex-limited factors [1,2,3,4]. Additionally, increasing the total number of piglets born has significantly increased this trait but also heightened piglet mortality [5]. Furthermore, an increase in the number of piglets often results in more piglets with low birth weights, which is associated with various long-term negative effects [6,7]. Genomic selection (GS) is a vital and effective strategy for determining litter size traits, production traits, and other economic traits, particularly for traits with low heritability or sex-limited influence such as litter size [8,9].

In GS, incorporating prior information, such as candidate genes or quantitative trait loci (QTLs) affecting traits, can enhance GS accuracy [10,11,12]. Genome-wide association studies (GWAS), a common method, have allowed researchers to discover QTLs associated with traits. Notable examples include the identification of *ESR1*, *FSH*, *EPOR*, *NR4A1*, *GNB2L1*, and *PRLR* variants associated with litter size traits in pigs [13]. To date, approximately 57,041 QTLs are associated with different traits in pigs, and more than 7568 QTLs are associated with reproduction traits in pigs (https://www.animalgenome.org/cgi-bin/QTLdb/SS/index; accessed on 5 April 2025) [13]. A general method for conducting GWAS involves fitting a univariate linear mixed model for marker association tests with a single phenotype or fitting a Bayesian sparse linear mixed model to estimating variance in phenotypes explained by single nucleotide polymorphism (SNP) heritability [14,15,16]. When ungenotyped animals have more phenotypic information, the common method for GWAS becomes difficult to perform, or there will be a loss of accuracy [17,18]. Compared to common methods, single-step GWAS (ssGWAS) can better integrate phenotypes, pedigrees, and genotyping data [19,20]. Additionally, ssGWAS can reduce noise, highlight the most significant peaks, and be adapted to complex ordinal models, such as repeatability models [18].

In many scenarios, whole-genome sequencing remains challenging, costly, and time-consuming in agricultural breeding. However, by simplifying genome sequencing, the cost of genotyping animals can be greatly reduced, and imputation-based strategies can significantly increase the density of genetic markers [21]. High-density genetic markers can effectively identify linkage disequilibrium (LD) in large populations and address issues related to genetic diversity and spatial structure [22]. Additionally, for non-model species without reference genomes, genotyping-by-sequencing (GBS) technology is well suited for revealing genetic diversity, genotyping, and genetic structure [23].

Large White pigs, one of the most commonly used pig breeds, are often employed as dam lines in modern commercial settings due to their advantages of large litters, high milk production, and strong maternal instincts. Therefore, the objective of this work was to perform high-density GS and ssGWAS using GBS technology in the Large White population to improve the accuracy of estimated breeding values and map the genomic regions associated with litter size traits.

## 2. Materials and Methods

### 2.1. Animals and Traits

The animals used in this study were sourced from 10 swine breeding herds in southern China, owned and managed by Wens Foodstuff Group Co., Ltd. (Yunfu, Guangdong, China). Data were collected from these herds between December 2010 and December 2020. The total number of litter sizes (total number born, TNB) observed was 170,027, which was available from 62,208 Large White sows. The study considered TNB, number of piglets born alive (NBA), number of healthy births (NHB), rate of NHB (rNHB), number of weak births (NWB), number of deformed fetuses (NDF), number of stillborn fetuses (NSB), mummified pigs (MUMM), and litter weight at birth (LBWT). Litters were removed from the data if they contained at most 3 piglets in the TNB, whereas litters of 22 piglets or larger were all considered “22”. Records of reproductive performance of 8 pairs or more were all considered “8”, and only 22,139 sows had a single observation. TNB is the number of all piglets born at the same birth, which equals the sum of NBA, NSB, and MUMM. NBA equals the sum of NHB, NWB, and NDF for all surviving piglets. NHB means that the birth weight is not less than 1.0 kg; in contrast, NWB means that the body weight is less than 1.0 kg, but NSB and NWB have no genetic defects. rNHB is the ratio of NHB to TNB. NDF refers to piglets with genetic defects, such as limb defects or reproductive system disorders. NSB refers to the number of dead piglets at birth or during pregnancy. In MUMM, a pig fetus dies or degenerates after the 35th day of pregnancy without abortion. LBWT is the total weight of all NBA within 24 h of birth.

### 2.2. Genotyping and Genotype Quality Control

The extraction of genomic DNA from ear tissue and the genotyping of whole-genome SNPs by the GBS technique have been explained in detail in previous studies. In detail, genomic DNA was digested with EcoRI/MspI restriction enzymes, ligated to barcoded adapters, and PCR-amplified. After Agilent 2100 fragment size selection, raw reads were quality-filtered (base quality ≥ 20; length ≥ 85 bp) and aligned to Sscrofa11.1 using TASSEL5.0, with genotype imputation via Beagle5.1 [24]. Quality control of the genotypes was performed using Plink (version 1.9 beta) [25], and the following criteria were used: SNPs with a minor allele frequency less than 0.01, SNPs that deviated significantly from Hardy–Weinberg equilibrium (HWE) (*p* < 10^−6^), SNPs with an individual call rate lower than 0.95, SNPs with a call rate lower than 0.99, and SNPs where the number of individuals in both homozygous genotypes (i.e., AA and BB) was less than 30 were excluded. After quality control, a total of 153,782 SNPs on autosomal chromosomes of Sus scrofa (SSCs) in 2096 pigs were available for further analysis.

### 2.3. Estimation of Genetic Parameters and Prediction Accuracy of GS

Genetic correlations and heritabilities between the nine litter size traits were estimated. The statistical model for describing the phenotypic data was as follows:

Y=Xb+Za+Wt+e,

where Y is the vector of phenotypic records, b is the vector of the fixed discrete effect of farrowing farm-year-month and parity, a is the vector of additive genotypic values (genomic estimated breeding value, GEBV), t is the vector of permanent environmental effects, e is the vector of residuals, and X, Z, and W are the known design matrices for b, a, and t, respectively. A univariate animal model and a bivariate animal model were used to calculate heritability and genetic correlation, respectively. Genetic parameters (heritabilities and correlations) were estimated using the BLUPF90 software suite (version 2.60). Genetic correlation magnitudes were classified as follows: very high—|r| ≥ 0.80; high—0.40 ≤ |r| < 0.80; low—0.2 < |r| < 0.40; very low—|r| < 0.2.

In this study, 10-fold cross validation was used to estimate the prediction performance of best linear unbiased prediction (BLUP) and single-step genomic BLUP (ssGBLUP) [26]. Each dataset was randomly divided into ten parts, nine of which were used as training datasets, and the remainder were used for validation. The prediction accuracy of the GEBV was as follows:

$$R_{g}=\sqrt{1-\mathrm{PEV}/\sigma_{a}^{2}},$$

where $R_{g}$ is the expected accuracy of predicting genetic values [27,28], PEV is the predictor error variance, and $\sigma_{a}^{2}$ is the additive genetic variance.

### 2.4. ssGWAS

The ssGWAS method is an improvement on the inverse of a numerator relationship matrix *A*^−1^, with *H*^−1^ replacing *A*^−1^ [29]:

$$H^{-1}=A^{-1}+\left[\begin{array}{cc} 0 & 0\\ 0 & G^{-1}-A_{22}^{-1} \end{array}\right],$$

where $G^{-1}$ is the inverse of a genomic relationship matrix and where $A_{22}^{-1}$ is a numerator relationship matrix for genotyped animals. The weighted genomic relationship matrix (G*) was as follows [30]:

$$G*=ZDZ^{\prime }\lambda$$

where *Z* is a matrix of gene content adjusted for allele frequencies, *D* is a weight matrix for SNPs (initially *D* = *I*), and λ is a variance ratio renormalizing constant. According to VanRaden et al. [31]. The calculated SNP effects and weights for the GWAS were as follows [19]:

(1) First, let *D* = *I*.
(2) Calculate *G*.
(3) The GEBV was calculated via ssGBLUP.
(4) The GEBV is converted to calculate the SNP effects ($\hat{u}$) as follows:

$$\hat{u}=\lambda DZ^{\prime }G^{-1}{\hat{a}}_{g},$$

where ${\hat{a}}_{g}$ is the GEBV for genotyped animals.

(5) The SNP weights are calculated as follows:
  $$d_{i}={\hat{u}}_{i}^{2}2p_{i}\left(1-p_{i}\right).$$
(6) The SNP weights are normalized to keep the total genetic variance constant.
(7) Loop to 2.

A previous study of this population demonstrated that LD (measured as r^2^ = 0.2) extends to approximately 0.3 Mb [24]. To ensure our window size captured haplotypic blocks reflecting the population’s LD structure, we adopted 0.3 Mb as the genomic window for ssGWAS. The proportion of genetic variance explained by the i-th window of successive SNPs was as follows [18]:

$$\frac{var\left(a_{i}\right)}{\sigma_{a}^{2}}\times 100\%=\frac{var\left(\sum_{j=1}^{m}Z_{j{\hat{u}}_{j}}\right)}{\sigma_{a}^{2}}\times 100\%,$$

where $a_{i}$ is the genetic value of the i-th window that consists of a region of successive SNPs located within 0.3 Mb, $\sigma_{a}^{2}$ is the total genetic variance, $Z_{j}$ is a vector genotype of the j-th SNP for all animals, ${\hat{u}}_{j}$ is the effect of the j-th SNP within the i-th window, and m is the number of SNPs within the i-th window. Overlapping windows were considered for variance calculations. Therefore, the top SNP was defined as contributing approximately equally to the 0.3 Mb-adjacent SNP window. The BLUPF90 family of programs was used to perform these analyses [32,33,34,35].

### 2.5. Identification of Candidate Genes and Analysis of Functional Enrichment

The candidate QTLs associated with litter size traits in the Large White population were defined as genomic windows that explained more than 1% of the genetic variance, and genes that were less than 0.3 Mb away from the potential SNPs were selected and identified as candidate genes via the Ensembl database (https://www.ensembl.org/; accessed on 5 April 2025) [36]. To better understand the biological processes and pathways associated with these candidate genes, Metascape [37] (https://metascape.org/; accessed on 5 April 2025) was used to perform Gene Ontology (GO) and Kyoto Encyclopedia of Genes and Genomes (KEGG) enrichment analyses. Metascape is based on the Homo sapiens (human) database and uses the default parameters. GO terms and KEGG pathways for which Fisher’s exact *p* value was less than 0.01 were considered significant.

## 3. Results

### 3.1. Descriptive Statistics for the Litter Size Traits

Statistics on the number of animals with or without records and genotypes in the Large White population are shown in Table 1. A total of 2096 pigs were genotyped, and 2036 of these also had phenotypic data recorded. Additionally, extensive genealogical databases were utilized in the study. Table 2 presents the descriptive statistics of the observed phenotypes. The coefficient of variation for the litter size traits ranged from 19% to 212%, indicating substantial phenotypic variation in the litter size traits within the Large White population. The kurtosis of TNB, NBA, NHB, and rNHB was close to zero, suggesting that these traits are suitable for animal models. In contrast, NDF, NSB, and MUMM may not be very suitable for animal models.

### 3.2. Heritabilities and Repeatabilities of the Litter Size Traits

The estimated variance components (additive genetic, permanent environmental, residual, and phenotypic variance), heritabilities, and repeatability of the litter size traits, along with their standard errors in the Large White population from the BLUP and ssGBLUP models, are shown in Table 3. No significant differences were observed between the two models. Heritability estimates for the litter size traits ranged from 0.01 to 0.06, indicating low heritability traits. Among the traits, TNB had the highest heritability (0.06), while NDF and MUMM had the lowest heritabilities (0.01). The repeatability estimates for the litter size traits ranged from 0.02 to 0.14. TNB and LBWT had the highest repeatability (0.14) and NDF had the lowest repeatability (0.02).

### 3.3. Genetic Correlations and Phenotypic Correlations Between the Litter Size Traits

The estimated genetic correlations and phenotypic correlations (lower and upper triangular sections, respectively), along with their standard errors in parentheses, between the nine litter size traits in the Large White population are shown in Table 4. High positive genetic correlations were estimated between TNB and other traits, except for rNHB. A very high positive genetic correlation of 0.90 was estimated between TNB and NBA. Negative correlations were estimated between rNHB and most traits (such as TNB, NBA, NWB, NDF, NSB, and MUMM), with a very high negative correlation of −0.78 between rNHB and NSB. Large differences were not observed between the genetic correlations and phenotypic correlations.

### 3.4. Prediction Accuracies of (G) EBV with BLUP and ssGBLUP in Litter Size Traits

The expected accuracies of predicting genetic values (Rg) across litter size traits for EBV and GEBV selection in the Large White population are shown in Table 5. The expected accuracies of BLUP ranged from 0.47 to 0.64, while the expected accuracies of ssGBLUP ranged from 0.50 to 0.70. The expected accuracies indicated that ssGBLUP had greater accuracy in genomic selection compared to traditional BLUP, with an increase ranging from 6.38% to 13.33%. Additionally, the expected accuracies of both BLUP and ssGBLUP showed very little variation (~0.01).

### 3.5. Summary of the ssGWAS Results for Litter Size Traits

**Figure 1:**
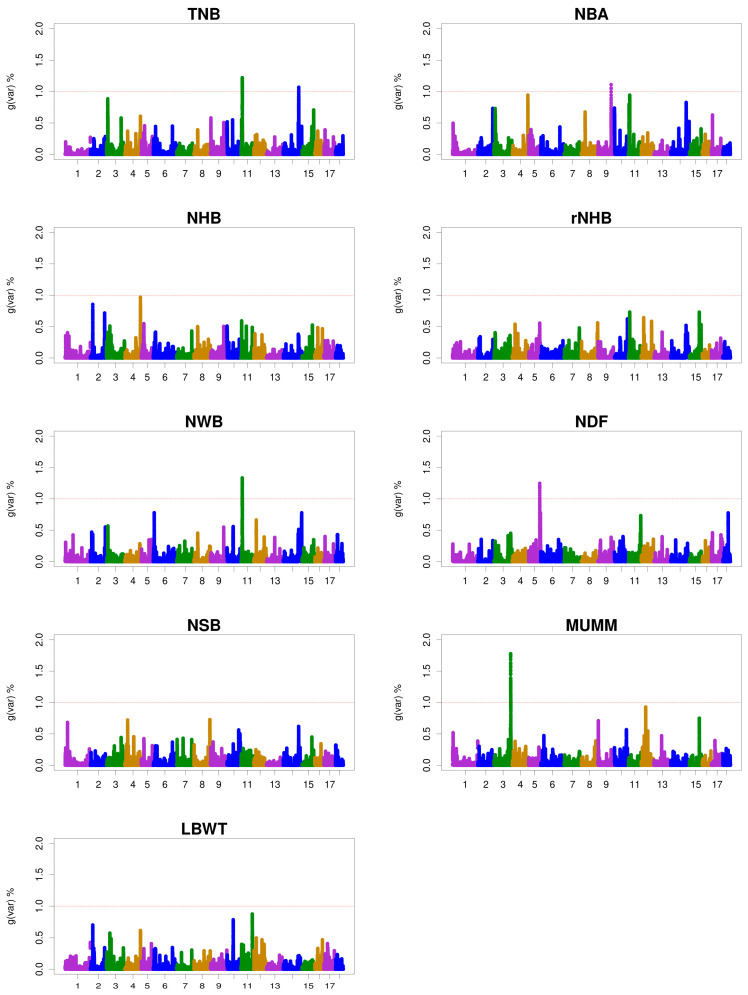
Manhattan plots for the percentage of genetic variance by 0.3 Mb window for litter size traits. gVar (%) represents the proportion of genetic variance explained by the 0.3 Mb window. The horizontal coordinate represents the chromosomes of pigs: total number born (TNB), number of piglets born alive (NBA), number of healthy births (NHB), rate of NHB (rNHB), number of weak births (NWB), number of deform fetus (NDF), number of stillborn (NSB), mummified pigs (MUMM), litter weight at birth (LBWT), and gVar (%) = the proportion of the genetic variance explained by the 0.3 Mb-adjacent SNP window.

The ssGWAS results were represented by the proportion of genetic variance explained by 0.3 Mb windows for the litter size traits (Figure 1). The genomic windows that explained more than 1.0% of the additive genetic variance in the litter size traits are shown in Table 6 and Tables S1–S5, along with the candidate genes near these genomic windows. In total, six genomic windows explaining between 1.07% and 1.77% of the additive genetic variance associated with litter size traits were identified. Additionally, two, one, one, one, and one genomic windows for TNB, NBA, NWB, NDF, and MUMM, respectively, were identified. No genomic windows were discovered for NHB, rNHB, NSB, and LBWT. In total, 26 candidate genes associated with litter size traits were identified, with the same candidate genes identified for TNB and NWB.

### 3.6. Functional Enrichment Analysis

Clusters with enriched terms related to litter size traits are shown in Table 7. The results of the GO analysis revealed that these genes were enriched in six functional categories, including signaling by receptor tyrosine kinases, cell morphogenesis involved in differentiation, cardiac muscle tissue development, negative regulation of cell differentiation, regulation of GTPase activity, and the RHO GTPase cycle (*p* < 0.01).

## 4. Discussion

### 4.1. Genetic Parameter Statistics

Heritability estimates for the litter size traits ranged from 0.01 to 0.06. Imboonta reported that the heritability of TNB in Thai Landrace pigs in the first four parities ranged from 0.02 to 0.11 [38]. Lundgren reported that the heritability of TNB in Landrace sows was 0.09 [39]. Camargo reported that the additive genetic variance and heritability of NBA in Landrace pigs were 0.90 and 0.09, respectively [40]. Damgaard reported that the heritability of NBA was 0.12 [41]. Lopez reported that heritability estimates for TNB ranged from 0.072 to 0.102, 0.090 to 0.099, and 0.109 to 0.121; from 0.087 to 0.110, 0.088 to 0.100, and 0.099 to 0.107 for NBA; and from 0.027 to 0.031, 0.050 to 0.053, and 0.073 to 0.081 for NSB in the Duroc, Landrace, and Yorkshire breeds, respectively [42]. Ogawa reported that the estimated heritabilities of TNB, NBA, NSB, and LBWT were 0.12, 0.12, 0.08, and 0.18, respectively, in Landrace pigs and 0.12, 0.10, 0.08, and 0.18, respectively, in Large White pigs [2]. These reports revealed low heritabilities in litter size traits. Compared to previous reports, our study found the lowest additive genetic variance and heritability.

Consistent with prior reports [43,44,45], TNB and NBA exhibit a very high genetic cor-relation (r ≥ 0.90), indicating similar selection effects. However, TNB-based selection is suboptimal due to its positive associations with NWB, NDF, NSB, and MUMM [46]. Alternatively, NHB and LBWT emerge as superior target traits given their contrasting correlation patterns: strong positive links with desirable traits (TNB/NBA/LBWT: NHB = 0.76/0.87/0.87; LBWT = 0.63/0.73/0.87); minimal associations with adverse outcomes (NWB/NDF/NSB/MUMM: NHB = 0.10/0.23/−0.11/0.11; LBWT = −0.04/0.23/−0.07/0.17). This supports Ogawa’s conclusion that LBWT optimizes NBA genetic improvement [2].

### 4.2. Genomic Prediction Accuracy

Genomic selection is widely regarded as a successful tool for genetic improvement in various livestock and plant species [47,48]. The single-step genomic best linear unbiased prediction (ssGBLUP) method has been extensively researched and applied [49]. In the present study, the accuracies of GEBV with ssGBLUP improved by 6.38% to 13.33% over the accuracies of the BLUP model for litter size traits. Teissier reported that the improvement between ssGBLUP and BLUP ranged from 5% to 7% [50]. However, the proportion of genetic variance explained by markers depends on the accuracy of GS. The size and family structure of the reference population may influence the accuracy of GS [51]. Additionally, identifying linkage disequilibrium depends on high-density genetic markers [52]. Thus, increasing the density of genetic markers may improve the accuracy of GS. Asora reported that accuracy increased as the number of markers and training sizes increased [53]. In this study, a high density of genetic markers and a large reference population significantly increased the accuracy of GS. In the future, we will continue to increase the size of the reference population and aim to improve or maintain the accuracy of GS.

### 4.3. Candidate Regions and Genes

**Figure 2:**
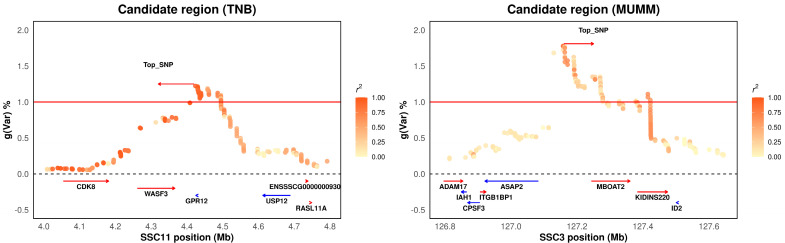
Candidate region plots illustrating the two major candidate regions on SSC11 and SSC3. The results are displayed for the total number born (TNB) at approximately 4.0–4.8 Mb on SSC11 (**left**) and for mummified pigs (MUMM) at 126.8–127.8 Mb on SSC3 (**right**). Various levels of linkage disequilibrium between the primary SNP and surrounding SNPs are represented by distinct colors. gVar (%) denotes the proportion of the genetic variance explained by the 0.3 Mb-adjacent SNP window.

In total, six genomic windows were identified. Among these significant windows, the region located at SSC11: 4.33–4.50 Mb explained 1.22% and 1.33% of the genetic variances for TNB and NWB, respectively. This region also explained relatively high genetic variance for NBA and rNHB (0.94%, 0.73%), with the top SNP located at SSC11: 4,423,721 bp. These findings indicate that these traits are highly genetically correlated. The gene adjacent to the top SNP (SSC11: 4,423,721 bp) was G protein-coupled receptor 12 (*GPR12*). Hinckley reported that *GPR12* was detected in the oocytes of Xenopus laevis, mice, and rats. Overexpression of *GPR12* in Xenopus laevis oocytes prevented progesterone-induced meiotic resumption. In reorganization systems, *GPR12* and *GPR3* appear to stimulate Gs activities in a ligand-independent fashion, suggesting that both *GPR3* and *GPR12* play key roles in controlling cAMP levels in the oocyte and in meiotic arrest [54]. Brown reported that *GPR12* may be involved in physiological processes such as the maintenance of oocyte meiotic arrest and brain development, as well as pathological conditions such as metastatic cancer [55]. Zhang reported that *GPR12* suppresses esophageal migration and promotes apoptosis in cancer and hypopharyngeal cancer [56]. Additionally, we determined the LD pattern of the SNPs in the region of SSC11: 4.0–4.8 Mb, which indicates a high level of LD between the top and nearby SNPs (Figure 2, left). These findings suggest potential selection signatures for litter size traits in the Large White population. Thus, we speculate that *GPR12* is a strong candidate gene for determining litter size traits because of its key role in the mechanisms of oocyte development.

The second most important genomic window was located at SSC3: 127.1–127.4 Mb, explaining 1.77% of the genetic variance in MUMM. The genes adjacent to the top SNP (SSC3: 127,157,661) were ArfGAP with SH3 domain, ankyrin repeat and PH domain 2 (*ASAP2*), and membrane-bound O-acyltransferase domain containing 2 (MBOAT2). Fujii reported that the novel driver gene *ASAP2* is a potential druggable target in pancreatic cancer [57]. Tekola-Ayele reported that *ASAP2* is associated with birth weight in humans and that the link between birth weight and placental DNA methylation is the opposite of what was previously reported in cord blood [58]. Tebani reported that *ASAP2* is associated with malignancies in different tissues and organs [59]. Zhou reported that circ-MBOAT2 modulates tumor development and glutamine catabolism via the miR-433-3p/GOT1 axis in pancreatic cancer, suggesting circ-MBOAT2 may be a therapeutic target for pancreatic cancer [60]. We also determined the LD pattern of the top SNPs in the region around SSC3: 126.8–127.8 Mb (Figure 2, right). We did not discover a high level of LD between the top and nearby SNPs. Therefore, while *ASAP2* and *MBOAT2* are strong candidate genes for determining litter size traits, further study is needed.

We also discovered other candidate genes associated with litter size traits. For example, treatment interventions targeting *CDK8* may have clinical benefits for beta-catenin-induced malignancies [61]. The *WASF3* gene promotes the invasion and metastasis of breast cancer cells that have undergone epithelial-to-mesenchymal transition, making it a potential target for inhibiting breast cancer cell infiltration and metastasis [62]. *USP12* acts as a regulator of neuron protein homeostasis and mHTT-mediated neurodegeneration [63]. The expression of *RASL11A* in primary colorectal tumors is lower than in normal mucosa [64]. Loss-of-function variants of biallelic *WDR11* are associated with intellectual disability and microcephaly [65]. *FGFR2* has been identified as a driver of intrahepatic disease [66]. *RABGAP1* is expressed mainly in follicular, granular, and epidermal cells [67]. High expression of *CACYBP* in hepatocellular carcinoma patients with poor prognosis is required for hepatocellular carcinoma cell growth both in vitro and in vivo [68]. The *MRPS-14* mutation can cause perinatal hypertrophic cardiomyopathy, lactate toxicity, growth retardation, deformities, and neurological involvement in neonates [69]. Loss of TNR results in nonprogressive neurodevelopmental disorders, with spasticity and transient visual impairment [70]. *TMTC2* variants are associated with familial bipolar sensorineural hearing loss and auditory neuropathy spectrum disorders [71]. The *KIDINS220* gene mutation, mediated by TrkA, plays a role in human brain ventriculomegaly [72]. These genes are associated with various human diseases, given the potential roles of the GO terms related to cell morphogenesis involved in differentiation. The GO enrichment results revealed that many genes are involved in cell morphogenesis and cardiac muscle tissue development.

## 5. Conclusions

In this study, the genetic parameters were estimated, and GS and ssGWAS were performed for the litter size traits of the Large White population. NHB and LBWT may be target traits, and their use may be beneficial for the genetic improvement of NBA. The accuracies of GEBV with ssGBLUP improved by 6.38% to 13.33% over the accuracies of the BLUP model in terms of litter size traits. ssGWAS results for these traits revealed a series of candidate genes, and *GPR12* is a strong candidate gene for determining litter size traits. These findings reveal the complexity of the genetic mechanisms underlying litter size traits and provide guidance for genetic improvement through genetic selection in the future.

## Figures and Tables

**Table 1:** Statistics on the number of animals with or without records and genotypes in the Large White population.

Statistic	Value
Number of animals with records	62,445
Number of animals with genotypes	2096
Number of animals with records or genotypes	62,505
Number of animals with genotypes and no records	60
Number of parents without records or genotypes	262,758
Total number of animals	325,263

**Table 2:** Descriptive statistics for litter size traits ^a^ in the Large White population.

Trait	n	Mean	SD	Min	Max	CV (100%)	Skewness	Kurtosis
TNB	170,027	13.77	3.51	4	22	25%	−0.28	0.03
NBA	166,880	12.21	3.34	3	21	27%	−0.29	0.02
NHB	166,310	10.52	2.92	2	20	28%	−0.39	0.23
rNHB	164,191	77.9	14.6	30	100	19%	−0.58	0.16
NWB	170,027	1.48	2.32	0	6	157%	1.05	0.61
NDF	170,027	0.19	0.26	0	3	137%	3.14	10.46
NSB	170,027	1.27	1.67	0	8	131%	1.91	4.09
MUMM	170,027	0.42	0.89	0	5	212%	2.8	8.97
LBWT	164,341	15.07	4.32	3	28	29%	−0.05	0.04

^a^ Total number born (TNB), number of piglets born alive (NBA), number of healthy births (NHB), rate of NHB (rNHB), number of weak births (NWB), number of deform fetus (NDF), number of stillborn (NSB), mummified pigs (MUMM), litter weight at birth (LBWT).

**Table 3:** Estimated variance components and heritabilities for litter size ^a^ traits in the Large White population.

Trait	Models	$\sigma_{a}^{2}$	$\sigma_{pe}^{2}$	$\sigma_{e}^{2}$	$\sigma_{p}^{2}$	H^2^	Re
TNB	BLUP	0.69 (0.05)	0.9 (0.04)	9.7 (0.04)	11.29 (0.04)	0.06 (0.01)	0.14 (0.01)
	ssGBLUP	0.68 (0.05)	0.92 (0.04)	9.7 (0.04)	11.30 (0.04)	0.06 (0.01)	0.14 (0.01)
NBA	BLUP	0.47 (0.04)	0.88 (0.04)	8.89 (0.04)	10.24 (0.04)	0.05 (0.01)	0.13 (0.01)
	ssGBLUP	0.46 (0.04)	0.89 (0.04)	8.89 (0.04)	10.24 (0.04)	0.04 (0.01)	0.13 (0.01)
NHB	BLUP	0.31 (0.03)	0.64 (0.03)	6.78 (0.03)	7.73 (0.03)	0.04 (0.01)	0.12 (0.01)
	ssGBLUP	0.31 (0.03)	0.64 (0.03)	6.78 (0.03)	7.74 (0.03)	0.04 (0.01)	0.12 (0.01)
rNHB	BLUP	8.49 (0.68)	9.48 (0.69)	180.19 (0.75)	198.15 (0.72)	0.04 (0.01)	0.09 (0.01)
	ssGBLUP	8.70 (0.68)	9.31 (0.68)	180.20 (0.75)	198.21 (0.73)	0.04 (0.01)	0.09 (0.01)
NWB	BLUP	0.10 (0.01)	0.11 (0.01)	1.96 (0.01)	2.17 (0.01)	0.04 (0.01)	0.10 (0.01)
	ssGBLUP	0.10 (0.01)	0.11 (0.01)	1.96 (0.01)	2.1 (0.01)	0.04 (0.01)	0.10 (0.01)
NDF	BLUP	0.01 (0.00)	0.01 (0.00)	0.25 (0.00)	0.2 (0.00)	0.01 (0.01)	0.02 (0.01)
	ssGBLUP	0.01 (0.00)	0.01 (0.00)	0.25 (0.00)	0.25 (0.00)	0.01 (0.01)	0.02 (0.01)
NSB	BLUP	0.12 (0.01)	0.08 (0.01)	2.35 (0.01)	2.5 (0.01)	0.05 (0.01)	0.08 (0.01)
	ssGBLUP	0.13 (0.01)	0.08 (0.01)	2.35 (0.01)	2.55 (0.01)	0.05 (0.01)	0.08 (0.01)
MUMM	BLUP	0.01 (0.00)	0.02 (0.00)	0.74 (0.00)	0.76 (0.00)	0.01 (0.01)	0.03 (0.01)
	ssGBLUP	0.01 (0.00)	0.02 (0.00)	0.74 (0.00)	0.76 (0.00)	0.01 (0.01)	0.03 (0.01)
LBWT	BLUP	0.81 (0.00)	1.34 (0.06)	13.38 (0.06)	15.53 (0.06)	0.05 (0.01)	0.14 (0.01)
	ssGBLUP	0.81 (0.06)	1.34 (0.06)	13.38 (0.06)	15.53 (0.06)	0.05 (0.01)	0.14 (0.01)

^a^ Total number born (TNB), number of piglets born alive (NBA), number of healthy births (NHB), the rate of NHB (rNHB), number of weak births (NWB), number of deform fetus (NDF), number of stillborn (NSB), mummified pigs (MUMM), litter weight at birth (LBWT). $\sigma_{a}^{2}$ = genetic variance; $\sigma_{pe}^{2}$ = permanent environmental variance; $\sigma_{e}^{2}$ = residual variance; $\sigma_{p}^{2}$ = phenotypic variance; H2 = heritability; and Re = repeatability. Standard errors in parentheses.

**Table 4:** Genetic correlations (below the diagonal) and phenotypic correlations (above the diagonal) for litter size traits ^a^ in the Large White population.

Trait	TNB	NBA	NHB	rNHB	NWB	NDF	NSB	MUMM	LBWT
TNB	\	0.84 (0.01)	0.70 (0.01)	−0.32 (0.01)	0.45 (0.01)	0.13 (0.01)	0.25 (0.01)	0.16 (0.01)	0.67 (0.01)
NBA	0.90 (0.01)	\	0.86 (0.01)	0.02 (0.01)	0.46 (0.01)	0.14 (0.01)	−0.20 (0.01)	−0.11 (0.01)	0.82 (0.01)
NHB	0.76 (0.02)	0.87 (0.01)	\	0.39 (0.01)	0.01 (0.01)	−0.02 (0.01)	−0.23 (0.01)	−0.12 (0.01)	0.85 (0.01)
rNHB	−0.45 (0.04)	−0.13 (0.06)	0.21 (0.05)	\	−0.57 (0.01)	−0.21 (0.01)	−0.66 (0.01)	−0.40 (0.01)	0.24 (0.01)
NWB	0.54 (0.04)	0.55 (0.03)	0.10 (0.05)	−0.69 (0.03)	\	0.01 (0.01)	−0.02 (0.01)	−0.04 (0.01)	0.15 (0.01)
NDF	0.36 (0.08)	0.38 (0.08)	0.23 (0.09)	−0.24 (0.09)	0.27 (0.08)	\	−0.02 (0.01)	−0.02 (0.01)	0.07 (0.01)
NSB	0.41 (0.04)	−0.04 (0.06)	−0.11 (0.05)	−0.78 (0.02)	0.10 (0.05)	0.35 (0.04)	\	0.08 (0.01)	−0.21 (0.01)
MUMM	0.49 (0.06)	0.21 (0.08)	0.11 (0.08)	−0.61 (0.05)	0.17 (0.07)	0.27 (0.11)	0.57 (0.06)	\	−0.11 (0.01)
LBWT	0.63 (0.03)	0.73 (0.02)	0.87 (0.01)	0.27 (0.05)	−0.04 (0.05)	0.23 (0.08)	−0.07 (0.05)	0.17 (0.08)	\

^a^ Total number born (TNB), number of piglets born alive (NBA), number of healthy births (NHB), the rate of NHB (rNHB), number of weak births (NWB), number of deform fetus (NDF), number of stillborn (NSB), mummified pigs (MUMM), and litter weight at birth (LBWT). Standard errors in parentheses.

**Table 5:** Accuracy of (G)EBV in the validation group using the BLUP and ssGBLUP methods.

Trait ^a^	BLUP-Rg	ssGBLUP-Rg	GBLUP vs. BLUP
			Increase %
TNB	0.64 (0.01)	0.70 (0.01)	9.38
NBA	0.61 (0.01)	0.68 (0.01)	11.48
NHB	0.60 (0.01)	0.68 (0.01)	13.33
rNHB	0.61 (0.01)	0.68 (0.01)	11.48
NWB	0.62 (0.01)	0.69 (0.01)	11.29
NDF	0.47 (0.01)	0.50 (0.01)	6.38
NSB	0.62 (0.01)	0.70 (0.01)	12.9
MUMM	0.52 (0.01)	0.57 (0.01)	9.62
LBWT	0.62 (0.01)	0.69 (0.01)	11.29

^a^ Total number born (TNB), number of piglets born alive (NBA), number of healthy births (NHB), rate of NHB (rNHB), number of weak births (NWB), number of deform fetus (NDF), number of stillborn (NSB), mummified pigs (MUMM), litter weight at birth (LBWT). Rg = expected accuracy of predicting genetic values. Standard errors in parentheses.

**Table 6:** Details of genomic regions and genes associated with litter size traits ^a^ in the Large White population.

Trait	SSC	Position (Mb)	gVar (%)	Number of SNPs	Top SNP Position	Candidate Genes
TNB	11	4.42–4.50	1.22	47	4,423,721	*CDK8*, *WASF3*, *GPR12*, *USP12*, *ENSSSCG00000009303*, *RASL11A*
TNB	14	130.94–130.95	1.07	4	130,945,803	*WDR11*, *ENSSSCG00000041462*, *ENSSSCG00000043365*, *FGFR2*
NBA	9	117.31–117.37	1.1	3	117,307,194	*RABGAP1L*, *CACYBP*, *MRPS14*, *TNN*, *KIAA0040*, *TNR*
NWB	11	4.33–4.48	1.33	43	4,423,721	*CDK8*, *WASF3*, *GPR12*, *USP12*, *ENSSSCG00000009303*, *RASL11A*
NDF	5	98.90–98.93	1.26	14	98,921,169	*TMTC2*, *METTL25*
MUMM	3	127.1–127.4	1.77	74	127,157,661	*ADAM17*, *IAH1*, *CPSF3*, *ITGB1BP1*, *ASAP2*, *MBOAT2*, *KIDINS220*, *ID2*

^a^ Total number born (TNB), number of piglets born alive (NBA), number of healthy births (NHB), the rate of NHB (rNHB), number of weak births (NWB), number of deform fetus (NDF), number of stillborn (NSB), mummified pigs (MUMM), litter weight at birth (LBWT). SSC = Sus scrofa chromosome; gVar (%) = the proportion of the genetic variance explained by the 0.3 Mb-adjacent-SNP window.

**Table 7:** The significant pathways for the positional candidate genes for litter size traits in Large White pigs.

GO	Description	Count	Percentage of All of the Provided Genes Related to Litter Size (%)	***p*-Values**
R-HSA-9006934	Signaling by Receptor Tyrosine Kinases	5	21.74	3.89 × 10^−5^
GO:0000904	cell morphogenesis involved in differentiation	5	21.74	1.51 × 10^−4^
GO:0048738	cardiac muscle tissue development	3	13.04	7.41 × 10^−4^
GO:0045596	negative regulation of cell differentiation	4	17.39	1.62 × 10^−3^
GO:0043087	regulation of GTPase activity	3	13.04	2.29 × 10^−3^
R-HSA-9012999	RHO GTPase cycle	3	13.04	4.57 × 10^−3^

Count = the number of genes in the user-provided lists with membership in the given ontology term.

## Data Availability

Genotype data were deposited into the European Variation Archive database under accession number PRJEB79359 and are available at the following URL: https://www.ebi.ac.uk/eva/?eva-study=PRJEB79359. The phenotypic data are not publicly accessible, as the populations consist of the nucleus herd of Wens Foodstuff Group Co., Ltd. However, this data is available from the corresponding author upon reasonable request.

## Supplementary Materials

The following supporting information can be downloaded at: https://www.mdpi.com/article/10.3390/ani15121724/s1. Table S1: The explained genetic variance of SNPs within significant windows for TNB; Table S2: The explained genetic variance of SNPs within significant windows for NBA; Table S3: The explained genetic variance of SNPs within significant windows for NWB; Table S4: The explained genetic variance of SNPs within significant windows for NDF; Table S5: The explained genetic variance of SNPs within significant windows for MUMM.
